# Lung disease associated with *filamin A* gene mutation: a case report

**DOI:** 10.1186/s13256-016-0871-1

**Published:** 2016-04-18

**Authors:** Safa Eltahir, Khalid S. Ahmad, Mohammed M. Al-Balawi, Hussien Bukhamsien, Khalid Al-Mobaireek, Wadha Alotaibi, Abdullah Al-Shamrani

**Affiliations:** King Fahad Medical City, P.O. Box 59046, Riyadh, 11525 Kingdom of Saudi Arabia

**Keywords:** *FLNA*, Lung disease, Angiogenesis, Pediatrics

## Abstract

**Background:**

Mutations in the gene encoding filamin A (*FLNA*) lead to diseases with high phenotypic diversity including periventricular nodular heterotopia, skeletal dysplasia, otopalatodigital spectrum disorders, cardiovascular abnormalities, and coagulopathy. *FLNA* mutations were recently found to be associated with lung disease. In this study, we report a novel *FLNA* gene associated with significant lung disease and unique angiogenesis.

**Case presentation:**

Here, we describe a 1-year-old Saudi female child with respiratory distress at birth. The child then had recurrent lower respiratory tract infections, bilateral lung emphysema with basal atelectasis, bronchospasm, pulmonary artery hypertension, and oxygen and mechanical ventilation dependency. Molecular testing showed a new pathogenic variant of one copy of c.3153dupC in exon 21 in the *FLNA* gene.

**Conclusions:**

Our data support previous reports in the literature that associate *FLNA* gene mutation and lung disease.

## Background

*Filamin A* is a 280-kD protein that crosslinks actin filaments into orthogonal networks in cortical cytoplasm and participates in the anchoring of membrane proteins for the actin cytoskeleton. Remodeling of the cytoskeleton is central to the modulation of cell shape and migration. Filamin A, encoded by the *FLNA* gene, is a widely expressed protein that regulates reorganization of the actin cytoskeleton by interacting with integrins, transmembrane receptor complexes, and second messengers, thus mutations in the gene encoding filamin A (*FLNA*) lead to diseases with high phenotypic diversity including the following: periventricular nodular heterotopia (PNH), skeletal dysplasia, otopalatodigital spectrum disorders such as Melnick-Needles syndrome and frontometaphyseal dysplasia [1–5], cardiovascular abnormalities that include patent ductus arteriosus (PDA), valvular disease, aortic root dilatation and aneurysms, Ehlers-Danlos syndrome-like phenotype [6–8], and coagulopathy with thrombocytopenia [9]. Recently, Masurel-Paulet *et al*. [1], de Wit and colleagues [10], and Lord *et al*. [11] reported associations between pulmonary diseases in both sexes with substantial heterogeneity in manifestations and lethality. Here, we report the case of a female child with a novel *FLNA* gene mutation who developed significant lung disease and unique angiogenesis.

## Case presentation

Our patient is a 1-year-old Saudi female child who was referred from another hospital after PDA ligation, left inguinal hernia repair, accidental fracture of her right ulna and radius, and prolonged mechanical ventilation for severe respiratory syncytial virus infection complicated by acute respiratory distress syndrome. The patient was sent to our hospital for further evaluation due to ongoing respiratory distress and hypoxemia. Our patient was the third child of nonconsanguineous Saudi parents and was born via cesarean section at 36 weeks gestation due to fetal distress. The child was admitted to the neonatal intensive care unit for 5 days because of respiratory distress and was ventilated for 36 hours. The mother has epilepsy and the father has been diagnosed with Behcet's disease. Since the age of 2 months, the child had multiple lengthy admissions in different hospitals (for 1 to 3 months at a time) for recurrent cyanotic events, respiratory distress, frequent choking with feeding, and significant vomiting. The child had accumulated the following diagnoses: severe gastroesophageal reflux disease (GERD), failure to thrive requiring prolonged nasogastric tube feeding, patent ductus arteriosus, pulmonary hypertension, anoxic convulsions, chronic lung disease with prolonged oxygen dependency, reversible bronchospasm, left external iliac vein thrombosis, and developmental delay. She had two prior prolonged stays at our institution.

**Fig. 1:**
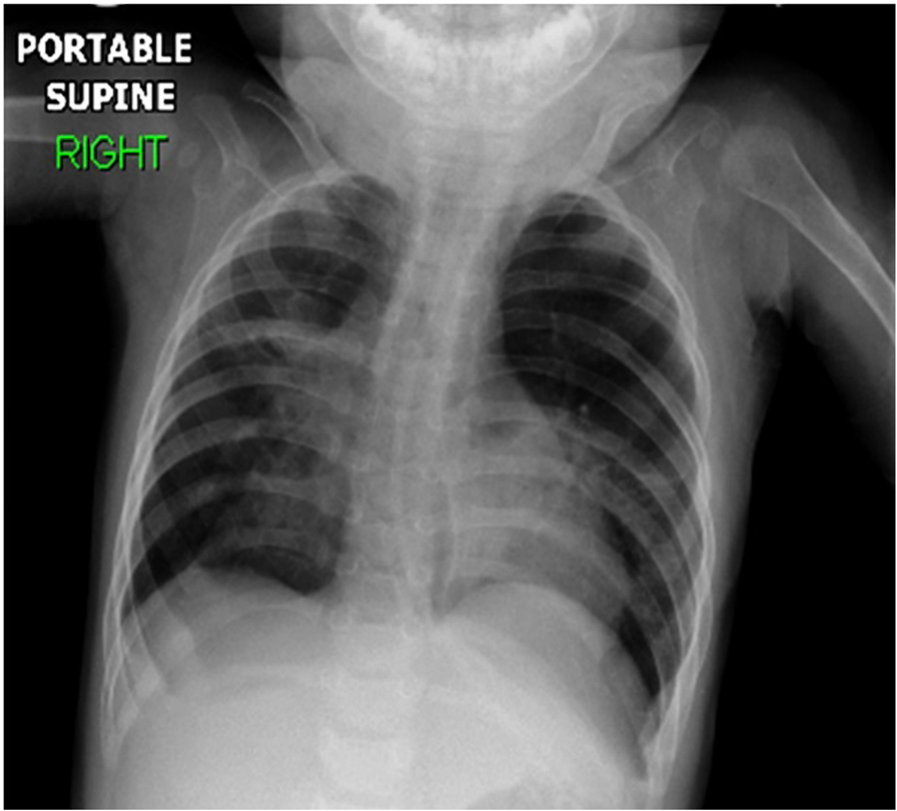
Chest X-ray anteroposterior view shows multiple subsegmental atelectasis, areas of air trapping and osteopenia

**Fig. 2:**
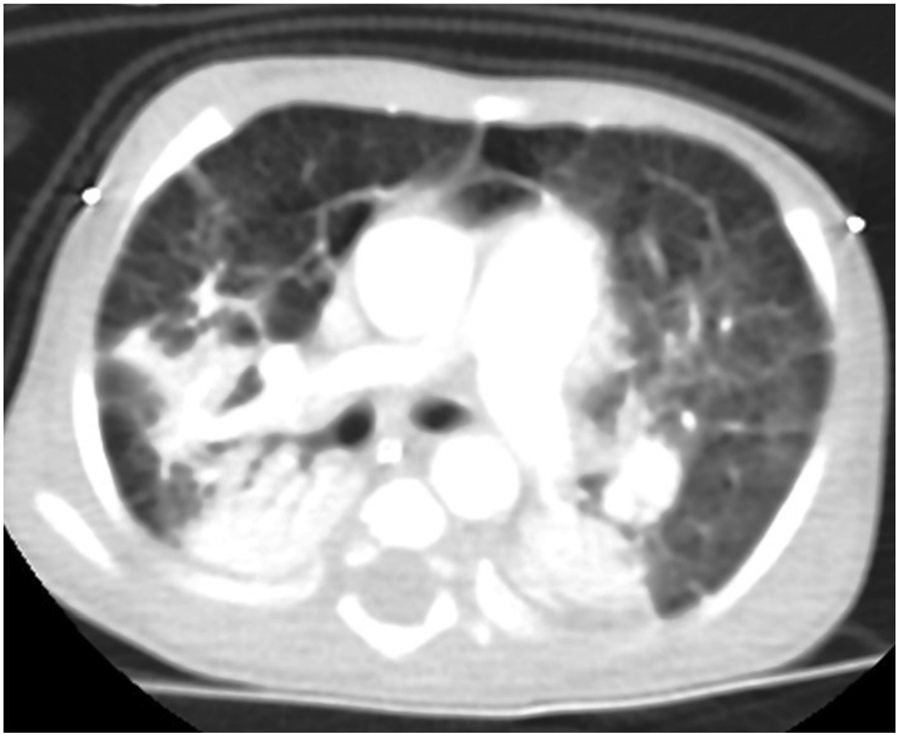
Computed tomography angiography axial view below bifurcation of carina shows basal lung atelectasis, lung hyperinflation, and enlarged main pulmonary artery

**Fig. 3:**
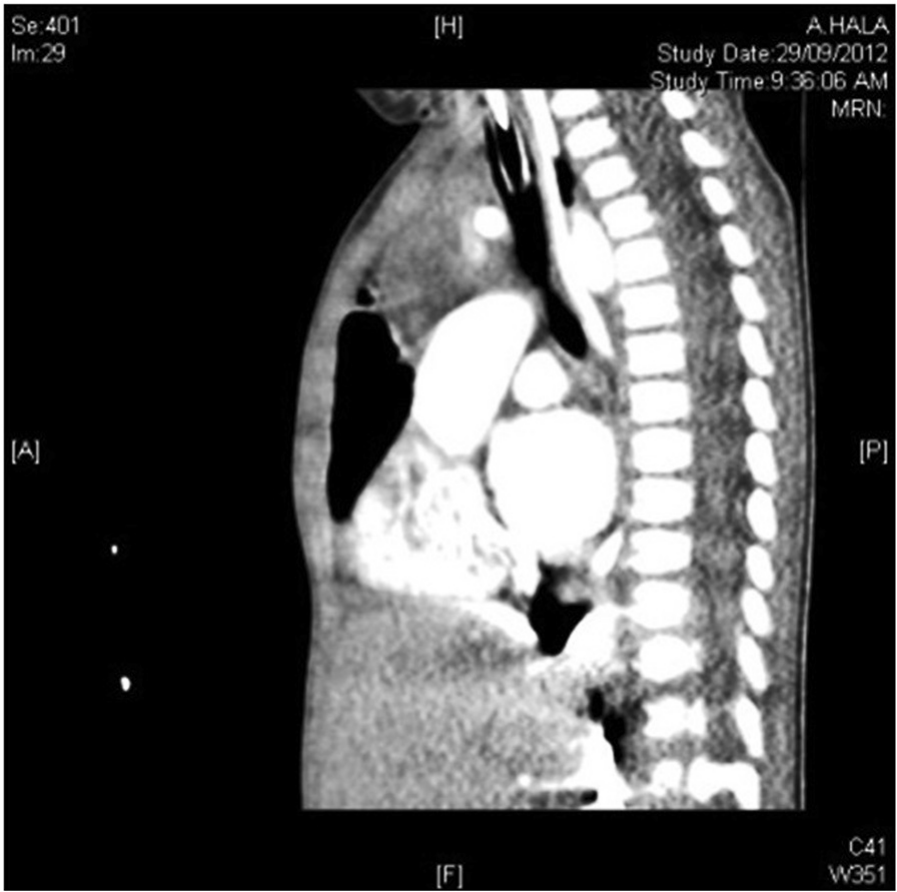
Computed tomography angiography sagittal view shows right aberrant subclavian artery posterior compression to the esophagus at level of T4 vertebra and minimal compression in the posterior trachea

**Fig. 4:**
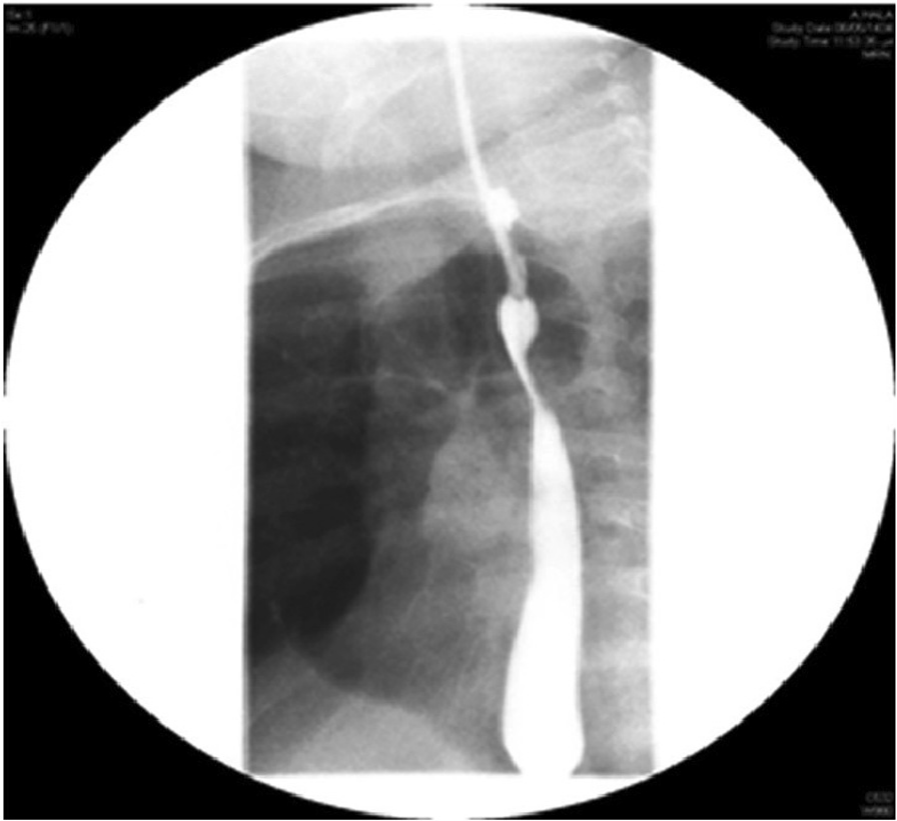
Barium meal shows external compression noted along posterior wall of proximal third of the thoracic esophagus causing significant narrowing of esophageal lumen

**Fig. 5:**
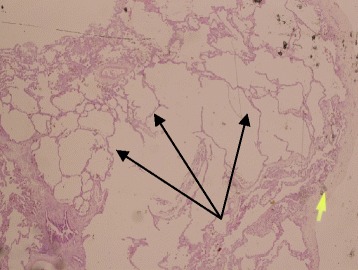
Alveolated lung parenchyma (black arrows) with alveolar simplification. *Small yellow arrow*: from the microscope that is probably not relevant

**Fig. 6:**
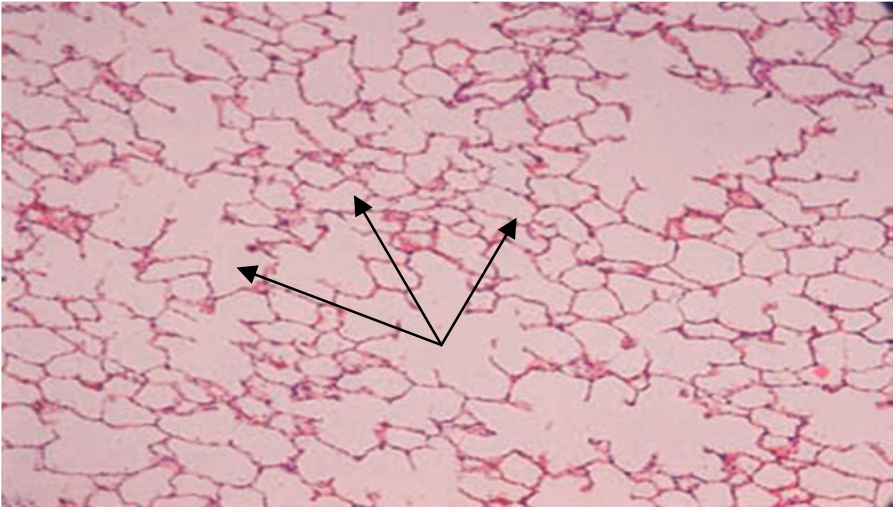
Normal alveolar septation

The first admission was due to rhinovirus infection and clinically diagnosed recurrent aspiration secondary to aberrant right subclavian artery (dysphagia lusoria) with prolonged oxygen therapy. The second admission was for respiratory failure that required prolonged intubation including high-frequency oscillatory ventilation complicated by recurrent lung atelectasis and right lung pneumothorax. She failed multiple trials of extubation and unfortunately died of cardiac arrest due to sepsis while receiving maximal supportive therapy. An initial physical examination during the first admission revealed a baby girl in poor health with the following clinical values: moderate respiratory distress and a respiratory rate of 70/min, heart rate of 144/min, blood pressure of 101/47 with saturation of 95% on 1.5 L/min, body weight of 5.9 kg (below the third percentile), and height of 69 cm (at the tenth percentile).The child had diminished breath sounds bilaterally with a prolonged expiratory phase, wheezing, and scattered crackles posteriorly, mild hypotonia, and significant hyperlaxity. Investigations showed normal sweat chloride level, and immune function testing was normal. A chest X-ray (Fig. 1) showed multiple subsegmental atelectasis and areas of air trapping. Computed tomography and angiography of her chest (Fig. 2) revealed bilateral lower lobe airspace disease, hyperinflation of the right middle lobe and left upper lobe including the lingual, and an enlarged main pulmonary artery. The sagittal view (Fig. 3) showed a right aberrant subclavian artery causing posterior compression to the esophagus at the level of the T4 vertebra and minimal compression in the posterior trachea. An echocardiogram showed no residual PDA or significant evidence of pulmonary hypertension. Barium administration (Fig. 4) showed external compression along the posterior wall of the proximal third of the esophagus, which was causing significant narrowing of the esophageal lumen. The pH probe showed no significant GERD. Upper gastrointestinal endoscopy showed there was a narrowed and compressed area located 18 cm into the esophagus at T4, and was identified with marked pulsation. The flexible bronchoscopy showed complete ring and narrowing of the lower third of the trachea. A lung biopsy (Fig. 5) showed alveolated lung parenchyma with alveolar simplification, in which alveoli do not show age-appropriate normal architecture, compared with the normal alveolar architecture (Fig. 6). There was no magnetic resonance imaging (MRI) of the brain because our patient's condition did not allow it.

### Gene analysis

Molecular testing for *FLNA*-related disorders in our patient showed a new pathogenic variant of one copy of c.3153dupC in exon 21 in the *FLNA* gene. This variant has not been reported in individuals with *FLNA*-related disorders but is expected to cause disease.

## Discussion

In this case report, we describe another confirmatory mutation in the gene encoding filamin A (*FLNA*) in a female child with lung disease. Our patient was a female child who was confirmed to have a pathogenic variant of the *FLNA* gene c.3153dupC. This mutation has not been reported in individuals with *FLNA*-related disorders but is expected to cause disease. Similar to other reported cases, our child also manifested clinical symptoms at an early age [10, 11]. She had respiratory distress at birth and then had recurrent lower respiratory tract infections/bronchiolitis, bilateral lung emphysema with basal atelectasis, bronchospasm, pulmonary artery hypertension, and long oxygen and mechanical ventilatory dependency after 2 months of age. A lung biopsy showed alveolated lung parenchyma with alveolar simplification similar to the finding by Lord *et al*. [11]. This child also manifested associated clinical features reported with other filamin gene mutations including patent ductus arteriosis (PDA), hypotonia, joint laxity, and developmental delay [10]. These clinical and histological features are similar to cases reported with the pulmonary phenotype of *FLNA* (Table 1). However, an unusual clinical manifestation in our case is severe reflux that caused recurrent aspiration symptoms and dysphagia lusoria (secondary to aberrant right subclavian artery that caused significant esophageal compression). This abnormal angiogenesis has been reported only in *FLNA*-null mice, which have vascular endothelial cells that show defective cell–cell contacts and adherens junctions. The result is abnormal angiogenesis with disorganized blood vessels and aberrant branching [7, 12]. There are other possible pulmonary phenotypes of *FLNA* gene mutation suggested due to its role in T cell activation, interleukin production [13], inflammatory signaling [14], and interaction with the cystic fibrosis transmembrane conductance regulator [15]. There has been no specific sensitivity to infections observed in *FLNA* patients [10]. However, our patient was noted to have severe prolonged courses of viral or bacterial infections. The pathogenic variant of *FLNA* gene mutation in our reported case is not confirmed to have association with periventricular nodular heterotopias (PNH), and our patient was too ill to complete brain MRI. The patient’s mother has epilepsy beginning in the third decade of life. The patient’s father has Behcet’s disease, which is highly consistent with other reports of PNH [8, 16, 17].

**Table 1:** Features of reported cases of filamin A mutation

	Masurel-Paulet *et al.* [1]	de Wit *et al.* [10]	Lord *et al*. [11]	This case
Genetic mutation	Mosaic nonsense *filamin A* mutation (c.994delG.P.K331X)	Missense *filamin A* mutation (c.220G>P.G74R )	Truncating *filamin A* mutation (c.5683G>T,p.G1895*)	Pathogenic variant (c.3153dupC) in exon 21 *filamin A* gene
Sex	Male	Female	Female	Female
Birth	Term with uncomplicated perinatal course	Term with uncomplicated perinatal course	Premature at 30 weeks mild respiratory distress resolved after 48 hours	Premature at 36 weeks with respiratory distress, needed ventilation for first 36 hours
Age at presentation	1.5 months	3 months	24 days	2 months
Pulmonary manifestation and pathology	Tachypnea, recurrent respiratory infections, asthma, prolonged oxygen dependence, lung atelectasis and lung cysts, tracheobronchomalacia, pulmonary arterial hypertension	Dyspnea, recurrent respiratory infections, prolonged oxygen dependence until 1 year and 7 months of age. Emphysema of right middle lobe, bronchomalacia of right bronchial tree	Tachypnea with desaturations, pulmonary arterial hypertension, oxygen dependence until 22 months of age bilateral pulmonary atelectasis and cysts. Tracheobronchomalacia	Recurrent cyanotic events, respiratory distress, episodes of choking and vomiting, with associated bronchospasm pulmonary arterial hypertension, prolonged oxygen and ventilator dependence until death at 15 months of age, bilateral pulmonary emphysema and basal atelectasis tracheal stenosis
Chest X-ray	Bilateral subsegmental atelectasis with hyperlucent areas in both mid zones		Cystic pulmonary lesions alternating with heterogeneous areas of atelectasis	Multiple subsegmental atelectasis and areas of air trapping
Chest CTscan	Widespread peribronchial thickening, subsegmental collapse, fluid within oblique fissure, eventration of the left hemidiaphragm	Severe lobar emphysema of right middle lobe with displacement of medistinal structures to left and compression of left upper lobe	Patchy ground-glass appearance with area of hyperinflation and cystic pulmonary lesions alternating with heterogeneous areas of atelectasis and thickening of interlobar septa	Bilateral lower lobe airspace diseases, hyperinflation of both upper lobes, enlarged main pulmonary artery, right aberrant subclavian artery, compressing esophagus and trachea
Surgery	Subtotal upper lobectomy at age of 8 months for lobar emphysema.	Lobectomy of right middle lobe for lobar emphysema		
Lung histology results	Panpulmonary emphysema with global absence of bronchial cartilage and hypertensive pulmonary vascular disease	Lung emphysema without inflammation	Mild to moderate chronic lung disease with associated alveolar simplification and pulmonary hypertension	Alveolated lung parenchyma with alveolar simplification
The associated nonpulmonary features	Periventricular nodular heterotopia with left cerebellar hemisphere hypoplasia and cisterna magna, truncal hypotonia, PDA, aortic root dilatation, bifid right urinary drainage system supraumbilical hernia, macrothrombocytes	Periventricular nodular heterotopia, with an enlarged retrocerebellar cyst, secundum atrial septal defect and coarctation of the aorta, hypotonia, severe hyperlaxity	Periventricular nodular heterotopia, secundum atrial septal defect, mild axial hypotonia	Suspected periventricular nodular heterotopia, PDA, angiogenesis causing dysphagia lusoria, hypotonia and joint laxity
Outcomes	Follow-up to 6 years	Follow-up to 3 years	Follow-up to 22 months	Death at 15 months

*CT* computed tomography, *PDA* patent ductus arteriosus

## Conclusions

In conclusion, we support the three previous reports in the literature, stating that there is an association between *FLNA* gene mutation and lung disease. The lung disease is a lethal condition likely manifested by early onset of pneumonia and recurrent bronchiolitis, subsequent asthma, pulmonary hypertension, and long-term oxygen dependency with underlying lung emphysema or lung cysts. *FLNA* mutation is also associated with airway anomalies such as tracheal stenosis (as in our patient) or tracheobronchomalacia. In addition, there are other diverse associated clinical manifestations.

## Consent

Written informed consent was obtained from the patient’s legal guardian for publication of this case report and any accompanying images. A copy of the written consent is available for review by the Editor-in-Chief of this journal.

## Abbreviations

FLNA: filamin A
GERD: gastroesophageal reflux disease
MRI: magnetic resonance imaging
PDA: patent ductus arteriosus
PNH: periventricular nodular heterotopia
